# Role of glymphatic system function in cognitive dysfunction among patients with bipolar disorder

**DOI:** 10.1093/ijnp/pyaf069

**Published:** 2026-02-02

**Authors:** Mu-Hong Chen, Ying-Jay Liou, Ju-Wei Hsu, Shih-Jen Tsai, Chiung-Chih Chang, Ya-Mei Bai

**Affiliations:** Department of Psychiatry, Taipei Veterans General Hospital, Taipei, Taiwan; Division of Psychiatry, School of Medicine, College of Medicine, National Yang Ming Chiao Tung University, Taipei, Taiwan; Institute of Brain Science, National Yang Ming Chiao Tung University, Taipei, Taiwan; Department of Psychiatry, Taipei Veterans General Hospital, Taipei, Taiwan; Division of Psychiatry, School of Medicine, College of Medicine, National Yang Ming Chiao Tung University, Taipei, Taiwan; Department of Psychiatry, Taipei Veterans General Hospital, Taipei, Taiwan; Division of Psychiatry, School of Medicine, College of Medicine, National Yang Ming Chiao Tung University, Taipei, Taiwan; Institute of Brain Science, National Yang Ming Chiao Tung University, Taipei, Taiwan; Department of Psychiatry, Taipei Veterans General Hospital, Taipei, Taiwan; Division of Psychiatry, School of Medicine, College of Medicine, National Yang Ming Chiao Tung University, Taipei, Taiwan; Institute of Brain Science, National Yang Ming Chiao Tung University, Taipei, Taiwan; Department of Neurology, Cognition and Aging Center, Institute for Translational Research in Biomedicine, Kaohsiung Chang Gung Memorial Hospital, Chang Gung University College of Medicine, Kaohsiung, Taiwan; Department of Psychiatry, Taipei Veterans General Hospital, Taipei, Taiwan; Division of Psychiatry, School of Medicine, College of Medicine, National Yang Ming Chiao Tung University, Taipei, Taiwan; Institute of Brain Science, National Yang Ming Chiao Tung University, Taipei, Taiwan

**Keywords:** glymphatic system function, bipolar disorder, cognition, analysis along the perivascular space index

## Abstract

**Objective:**

Increasing evidence has suggested a potential role of glymphatic system function in the pathomechanisms underlying mood disorders and related cognitive impairment. However, this association in bipolar disorder remained unclear.

**Design, Participants, and Measures:**

In all, 185 patients (mean age ~ 30 years) with bipolar disorder and 95 healthy individuals were enrolled for the assessment of the go/no-go task and the digit symbol substitution test (DSST). The Analysis Along the Perivascular Space (ALPS) index, an indicator of glymphatic system activity, was measured using diffusion tensor imaging within diffusion magnetic resonance imaging. All participants were divided into two subgroups (< vs. ≥ mean level) based on the mean ALPS index level. Additionally, participants were further categorized into quartiles based on ALPS index levels, ranging from Q1 (highest) to Q4 (lowest).

**Results:**

Generalized linear model, after adjusting for age, sex, education years, and clinical symptoms, found no difference in the ALPS index levels between patients and controls. Patients with lower ALPS index levels—that is, those with values below the mean or in the lowest quartile (Q4)—performed the worst on the DSST and the go/no-go task.

**Conclusions and Relevance:**

Glymphatic system dysfunction appeared to play a critical role in cognitive impairment among patients with bipolar disorder, but not in healthy individuals. Further research is warranted to clarify the specific pathomechanisms underlying this bipolar disorder–specific association between glymphatic system dysfunction and cognitive deficits.

Significance Statement The glymphatic system clears neurotoxic waste from the central nervous system and is most active during sleep. In this study, glymphatic system dysfunction was associated with cognitive impairment in individuals with bipolar disorder, despite no significant between-group difference in the Analysis Along the Perivascular Space index (a proxy of glymphatic system function) relative to healthy controls. Future research should investigate whether improving glymphatic system function—e.g., through sleep-focused interventions—can mitigate cognitive deficits in bipolar disorder.

## INTRODUCTION

The glymphatic system, first described in 2012, is a brain-wide perivascular network formed by astroglial cells that facilitates the clearance of interstitial waste products and neurotoxic substances (eg, amyloid-β) from the vertebrate central nervous system.^1–3^ This system functions primarily during sleep, promoting the influx of cerebrospinal fluid (CSF) along periarterial spaces, where it mixes with interstitial fluid to remove metabolic waste via astroglial aquaporin-4 channels.^1–3^ Dysfunction of the glymphatic system has been implicated in the pathogenesis of various neurodegenerative disorders, including Alzheimer’s disease, Parkinson’s disease, and amyotrophic lateral sclerosis.^1–3^

Given the critical role of sleep in maintaining glymphatic system function,^1–3^ accumulating evidence has demonstrated a significant association between sleep disruption and glymphatic system dysfunction.^4^^,^^5^ Glymphatic system activity is commonly inferred using the Analysis Along the Perivascular Space (ALPS) index, an indirect imaging biomarker derived from diffusion tensor imaging (DTI) within diffusion magnetic resonance imaging (MRI).^4^^,^^5^ In the Human Connectome Project study, which included 317 individuals with sleep disruption and 515 healthy controls, the ALPS index was significantly lower in those with sleep disruption compared to healthy individuals.^4^ Saito et al. further reported an inverse correlation between the ALPS index and Pittsburgh Sleep Quality Index total scores, indicating that more severe sleep disturbances were associated with greater glymphatic dysfunction (ie, lower ALPS index).^4^ Despite the fact that sleep dysregulation is a core feature of bipolar disorder, research investigating the relationship between glymphatic system function and bipolar disorder remains limited.^6^ In September 2024, Ueda et al. provided the first evidence suggesting a potential role for glymphatic system dysfunction in the pathophysiology of bipolar disorder. Their study involved a relatively small sample of 58 middle-aged patients with bipolar disorder (mean age ~ 48.8 years) and 66 healthy controls.^6^ Although no significant differences in the ALPS index were observed between groups, the study revealed an inverse relationship between the ALPS index and illness duration in patients with bipolar disorder.^6^ These preliminary findings underscore the need for further investigation in larger samples to validate and expand upon the proposed association between glymphatic system dysfunction and bipolar disorder.

In addition to the established link between sleep disturbance and glymphatic system function, emerging evidence suggests that glymphatic dysfunction may play a critical role in cognitive impairment, potentially contributing to deficits in working memory, attention, inhibitory control, and executive function.^7–9^ For instance, the Framingham Heart Study, which included 2682 middle-aged individuals (mean age ~ 56 years), demonstrated that higher ALPS index values were significantly associated with better executive function.^8^ Similarly, Yu et al. found a positive correlation between ALPS index values and total scores on the Montreal Cognitive Assessment in middle-aged individuals (~60 years) with type 2 diabetes mellitus.^9^ Although cognitive dysfunction—particularly impairments in inhibitory control and executive function—is a well-documented feature of bipolar disorder,^10^^,^^11^ no studies to date have specifically examined the association between glymphatic system function and cognitive performance in individuals with bipolar disorder. This gap highlights the need for further research to elucidate the potential contribution of glymphatic system dysfunction to the cognitive deficits observed in this population.

The present study enrolled 185 adult patients aged 20 to 59 years with a diagnosis of bipolar disorder, along with 95 age- and sex-matched healthy individuals, to evaluate the ALPS index and assess inhibitory control and executive functions. We hypothesized that patients with bipolar disorder would exhibit lower ALPS index levels compared to healthy individuals, reflecting impaired glymphatic system function. Furthermore, we hypothesized that, within the patient group, lower ALPS index levels would be associated with greater deficits in inhibitory control and executive functioning.

## METHODS

### Participants

The present study enrolled adult patients aged between 20 and 59 years who were diagnosed with bipolar disorder based on the Diagnostic and Statistical Manual of Mental Disorders, Fifth Edition. Patients who had lifetime diagnoses of schizophrenia, alcohol and substance use disorders, and neurodevelopmental disorders were excluded in the present study. All patients scored ≤4 on the Clinical Global Impression rating scale and complied with the study procedure. The present study also enrolled age- and sex-matched healthy individuals who had no diagnosis of any psychiatric disorders as the control group. All participants had no diagnosis of any major physical conditions, including neurovascular diseases, epilepsy, autoimmune diseases, and major cognitive disorders. Clinical symptoms were assessed using the Young Mania Rating Scale (YMRS) and the 17-item Hamilton Depression Rating Scale (HDRS).^12^^,^^13^ All participants underwent the MRI assessment. According to the evidence that the effect sizes of glymphatic system dysfunction between clinical and healthy subjects ranged between 0.6 and 0.8,^14–16^ G-power analysis indicated the optimal sample size of ≥148 (74 for each group) using the effect size = 0.6, α = 0.05, and power = 0.95. The study was conducted in accordance with the Declaration of Helsinki and was approved by the Institutional Review Board of Taipei Veterans General Hospital. Written informed consent was obtained from all participants prior to their inclusion in the study.

### Measurement of the ALPS Index

The detail of the ALPS index measurement has been reported in the previous study.^7^ All MRI images were acquired on a 3-Tesla scanner (GE Healthcare Life Sciences, Little Chalfont, UK) equipped with a quadrature head coil. High-resolution T1-weighted anatomical images were obtained using a 3D magnetization-prepared rapid acquisition gradient echo sequence using the following parameters: repetition time (TR) = 12.2 ms, echo time (TE) = 5.2 ms, flip angle = 12°, 168 axial slices, field of view (FOV) = 256 × 256 mm, matrix size = 256 × 256, and slice thickness = 1 mm. DTI was acquired using a single-shot spin-echo echo-planar imaging sequence. Diffusion-weighted images were obtained with b = 1000 s/mm^2^, using 64 noncollinear diffusion gradient directions. Axial images were acquired using the following parameters: TR/TE = 8800/91 ms; 70 axial slices, flip angle = 90°, slice thickness = 2.2 mm; FOV = 256 × 256 mm. Briefly speaking, ALPS index was computed using DTI to assess glymphatic function. Preprocessing of DTI data included (1) denoising based on Marchenko-Pastur principal component analysis, (2) removal of Gibbs ringing artifacts, (3) correction for motion and distortion, and (4) bias field correction using the b = 0 image as a reference. Diffusivity maps along the x-axis (Dxx, right–left), y-axis (Dyy, anterior–posterior), and z-axis (Dzz, superior–inferior) were extracted. The ALPS index was derived by measuring diffusivities orthogonal to projection and association fibers adjacent to the lateral ventricle body. The corticofugal corona radiata tract fibers represented projection fibers, and the superior longitudinal fasciculus represented association fibers, based on the JHU DTI-based white matter atlas (https://neurovault.org/collections/264/). A 5-mm thickness mask confined the selected fibers along the lateral ventricle body, and transformations between Montreal Neurological Institute (MNI)-152 space and native fractional anisotropy (FA) space were applied for localization. The ALPS index was calculated using the following formula: ALPS index = mean (Dyy_projection_, Dzz_association_)/mean (Dxx_projection_, Dxx_association_). Higher ALPS index values indicate better glymphatic function, reflecting more efficient perivascular fluid transport in the brain.^7^ Finally, all participants (mean age ~ 33 years) were divided into two subgroups (< vs. ≥ mean level) based on the mean ALPS index level (1.061), which closely aligned with the mean reference level (~1.06) of the ALPS index for individuals in their 30s from Hsiao et al.’s study of 433 Taiwanese individuals aged 10 to 80 years.^7^ In addition, all participants were further divided into quartiles based on the ALPS index level: Q1 (the highest) to Q4 (the lowest).

### Assessment of Cognitive Function

The go/no-go task and the digit symbol substitution test (DSST) were used for the cognitive function assessment.^17^^,^^18^ The go/no-go task is a cognitive paradigm assessing response inhibition and executive control. Participants respond to “Go (×)” stimuli while withholding responses to “No-Go (+)” stimuli. We calculated the errors, mean reaction time (ms), and standard deviation (SD) of the mean reaction time of the go/no-go task. The go/no-go task performance reflects impulse control and attentional regulation. Furthermore, the participants are instructed to quickly and accurately fill in the corresponding symbol for each digit using the key table in the DSST. They match symbols to digits based on a reference key within 120 seconds. The DSST performance reflects processing speed, working memory, and attention. The go/no-go task and the DSST were commonly used in our previous studies.^19–21^

### Statistical Analysis

For between-group comparisons, the F-test was used for continuous variables and Pearson’s test was used for categorical variables. Generalized linear models (GLMs) with adjustment of demographic data (age, sex, and education years) and clinical symptoms (HDRS, YMRS) were performed to assess the ALPS index between patients with bipolar disorder and healthy individuals. Additional GLMs with adjustment of demographic data (age, sex, and education years) and clinical symptoms (HDRS, YMRS) were conducted to evaluate the effects of group (bipolar disorder vs. control), the ALPS index (< vs. ≥ mean level and Q1 vs. Q4), and their interactions on cognitive function. Finally, given the crucial role of insomnia symptoms in the glymphatic system function,^4^^,^^5^ we further performed a correlation analysis between the ALPS index and insomnia symptoms, indicated by the HDRS insomnia symptom subtotal scores (the sums of HDRS items 4, 5, and 6), after adjusting for demographic data in patients with bipolar disorder. A two-tailed *P* value of less than 0.05 was considered statistically significant. All data processing and statistical analyses were performed using the SPSS version 17 software (SPSS Inc., Chicago, IL).

## RESULTS

In all, 185 patients with bipolar disorder and 95 healthy individuals were enrolled in the present study, with a mean age of ~33 years (Table 1). Sex (*P*=.146) and the ALPS index levels (*P*=.533) did not differ between the two groups (Table 1). Patients with bipolar disorder had shorter years in the education than did the control group (*P*<.001) (Table 1). The mean scores of the HDRS and YMRS were 9.77 ± 6.60 and 4.18 ± 5.37, respectively, among patients with bipolar disorder (Table 1).

**Table 1:** Demographic and clinical characteristics between groups.

	Patients with bipolar disorder (*n* = 185)	Heathy individuals (*n* = 95)	*P*-value
**Age (years, SD)**	34.63 (11.27)	32.51 (9.41)	.116
**Sex (n, %)**			.146
** Male**	59 (31.9)	39 (41.1)	
** Female**	126 (68.1)	56 (58.9)	
**Education (years, SD)**	14.05 (2.95)	15.92 (2.03)	<.001
**Duration of illness (years)**	7.52 (8.82)		
**ALPS index (SD, n, %)**	1.06 (0.06)	1.06 (0.05)	.533
** < mean**	98 (53.0)	51 (53.7)	
** ≥ mean**	87 (47.0)	44 (46.3)	
** Q1 (highest)**	47 (25.4)	23 (24.2)	
** Q2**	47 (25.4)	23 (24.2)	
** Q3**	45 (24.3)	25 (26.3)	
** Q4 (lowest)**	46 (24.9)	24 (25.3)	
**Clinical state (n, %)**			
** Euthymic state**	70 (37.8)		
** Depressive state**	87 (47.1)		
** Manic/hypomanic state**	28 (15.1)		
**Clinical symptoms (SD)**			
** YMRS**	4.18 (5.37)	0.00 (0.00)	<.001
** HDRS**	9.77 (6.60)	0.00 (0.00)	<.001
** Insomnia subtotal scores**	1.24 (1.67)	0.00 (0.00)	<.001
**Psychotropic medications (n, %)**			
** Mood stabilizers**	80 (43.2)		
** Atypical antipsychotics**	103 (55.7)		
** Antidepressants**	46 (24.9)		

Abbreviations: ALPS: analysis along the perivascular space; HDRS: 17-item Hamilton Depression Rating Scale; SD: standard deviation; YMRS: Young Mania Rating Scale.

GLM, after adjusting for age, sex, education years, and HDRS and YMRS scores, found no difference in the ALPS index levels between the bipolar disorder and control groups (*P*=.480). In the entire sample, GLMs revealed a significant interactive effect of diagnosis and ALPS index (< vs. ≥ mean level) on DSST performance (*P*=.002); in the Q1 and Q4 sample, GLMs further demonstrated a significant interactive effect of diagnosis and ALPS index (Q1 vs. Q4) on mean reaction time (*P*=.005) and the SD of mean reaction time (*P*=.022) in the go/no-go task (Table 2). The significant effect of the ALPS index (*P*=.009; 0.019) was noted in the DSST performance both in the entire sample (< vs. ≥ mean level) and in the Q1 and Q4 (Q1 vs. Q4) sample (Table 2). Additionally, a significant effect of diagnosis was found in the SD of mean reaction time in the go/no-go task (*P*=.033) in the entire sample. The effect of diagnosis was also significant in DSST performance, both in the entire sample (*P*<.001) and in the Q1 and Q4 sample (*P*<.001) (Table 2). In addition, we found no association between the ALPS index and insomnia symptoms (B = 0.003, *P*=.296) in patients with bipolar disorder.

**Table 2:** Generalized linear models with adjustment of age, sex, education, and clinical symptoms (YMRS and HDRS) for associations between diagnosis, glymphatic system function, and cognitive functions.

	All sample (n = 280)				Q1 and Q4 sample (n = 140)		
	Wald χ^2^	df	*P*-value		Wald χ^2^	df	*P*-value
	Go/No-go task: errors						
**Diagnosis (D; bipolar disorder vs. control)**	0.00	1	.956	Diagnosis (D; bipolar disorder vs. control)	0.07	1	.789
**ALPS index (< vs. ≥ mean)**	1.04	1	.307	ALPS index (Q1 vs. Q4)	3.37	1	.067
D*ALPS index	2.28	1	.131	D*ALPS index	0.65	1	.424
	Go/No-go task: mean time (ms)						
**Diagnosis (D; bipolar disorder vs. control)**	4.81	1	.028	Diagnosis (D; bipolar disorder vs. control)	1.13	1	.289
**ALPS index (< vs. ≥ mean)**	1.00	1	.316	ALPS index (Q1 vs. Q4)	0.69	1	.408
D*ALPS index	3.49	1	.062	D*ALPS index	7.95	1	**.005**
	Go/No-go task: SD of mean time						
**Diagnosis (D; bipolar disorder vs. control)**	4.56	1	**.033**	Diagnosis (D; bipolar disorder vs. control)	0.935	1	.334
**ALPS index (< vs. ≥ mean)**	0.90	1	.344	ALPS index (Q1 vs. Q4)	0.795	1	.373
D*ALPS index	1.64	1	.201	D*ALPS index	5.242	1	**.022**
	DSST						
**Diagnosis (D; bipolar disorder vs. control)**	14.10	1	**<.001**	Diagnosis (D; bipolar disorder vs. control)	15.493	1	**<.001**
**ALPS index (< vs. ≥ mean)**	6.88	1	**.009**	ALPS index (Q1 vs. Q4)	5.480	1	**.019**
D*ALPS index	9.62	1	**.002**	D*ALPS index	2.950	1	.086

**Bold** type indicates the statistical significance (*P*<.05).

Abbreviations: ALPS: analysis along the perivascular space; DSST: digit symbol substitution test. HDRS: 17-item Hamilton Depression Rating Scale; SD: standard deviation; YMRS: Young Mania Rating Scale.

**Figure 1:**
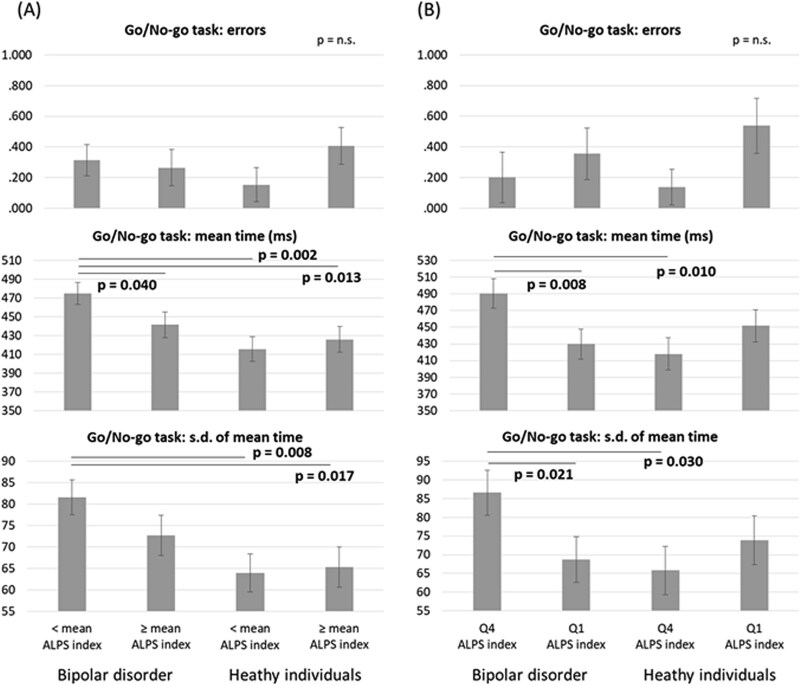
Estimated measures of the go/no-go task according to the GLM model for the interaction between diagnosis and analysis along the perivascular space index. (A) all sample; (B) Q1 and Q4 sample. GLM: Generalized linear model; SD: Standard deviation. Note: Adjusting for age, sex, education, and clinical symptoms.

**Figure 2:**
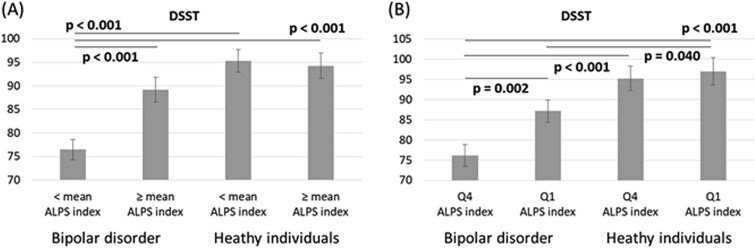
Estimated measures of the DSST according to the GLM model for the interaction between diagnosis and analysis along the perivascular space index. (A) All sample; (B) Q1 and Q4 sample. GLM: Generalized linear model; DSST: Digit symbol substitution test. Note: Adjusting for age, sex, education, and clinical symptoms.

Finally, Figs 1 and 2 illustrated the estimated measures of the go/no-go task and the DSST, depicting the interaction effects between diagnosis and ALPS index. Both in the entire sample and in the Q1 and Q4 sample, patients with bipolar disorder who had a low ALPS index level—both those with an ALPS index level < mean and those in Q4—exhibited the poorest performance compared with the other three groups. This included the longest mean reaction time and the greatest SD of mean reaction time in the go/no-go task, as well as the lowest number of correct responses in the DSST (Figs.1 and 2).

## DISCUSSION

Our findings did not support the initial hypothesis that patients with bipolar disorder would exhibit lower ALPS index levels compared to healthy individuals. However, we found that patients with lower ALPS index levels—specifically those with values below the mean or within the lowest quartile (Q4)—demonstrated the poorest performance on the go/no-go task and the DSST. These results may suggest that cognitive impairments in domains such as inhibitory control, processing speed, attention, and executive function were present primarily in patients with glymphatic system dysfunction, rather than in those with relatively preserved glymphatic system function.

Several preclinical studies have suggested an association between glymphatic system dysfunction and mood disorders.^22^^,^^23^ Xia et al. demonstrated impaired glymphatic pathway circulation in mice subjected to chronic unpredictable mild stress (CUMS), an established animal model of depression, and suggested that glymphatic system dysfunction was particularly associated with anhedonic symptoms—a clinical hallmark of bipolar depression.^22^ They further observed that treatment with fluoxetine normalized glymphatic function in CUMS-treated mice.^22^ Similarly, Liu et al. reported significantly reduced glymphatic system function in the cortex, subcortex, and hippocampus, and found associations between these reductions and cognitive deficits as measured by the Morris water maze (spatial cognition) and the novel object recognition test (memory) in CUMS-treated mice.^23^ However, a small-sample study involving 58 middle-aged patients (mean age ~ 50 years) with bipolar disorder and 66 healthy controls found no statistically significant difference in the ALPS index.^6^ This result aligned with our findings from a larger sample, which showed no significant difference in the ALPS index between young adult patients (mean age ~ 30 years) with bipolar disorder and healthy controls. Interestingly, our observation that patients with lower ALPS index values performed worst on the go/no-go task and the DSST may echo Liu et al.’s findings, in which CUMS-treated mice exhibited memory and spatial cognition impairments alongside decreased levels of aquaporin-4—a marker of glymphatic system function.^23^ Further investigation is needed to clarify the inconsistency in glymphatic system dysfunction between the clinical and preclinical studies and to elucidate the potential role of the glymphatic system in the pathomechanisms underlying bipolar disorder.

The effect of glymphatic system function on cognitive function between adult patients (mean age ~ 30 years in the present study) and healthy controls warranted discussion. Specifically, our study found that glymphatic system function-related differences in cognitive function were only noted in patients with bipolar disorder but not in healthy controls. Previous studies have shown that glymphatic function decreases progressively with age, particularly after 65 years, and mediates the relationship between aging and cognitive decline.^7^^,^^24^ Increasing evidence reported the crucial role of the glymphatic system in the clearance of amyloid-β and tau, which commonly start to accumulate after age 45-50 years.^25^^,^^26^ However, studies involving an association between glymphatic system function and cognition in healthy young adults are lacking. Whether cognitive impairment may not be evident in healthy young adults with suboptimal glymphatic system function remained unknown. A possible hypothesis to explain the glymphatic system function-related differences in cognitive performance between patients and healthy individuals is that glymphatic system dysfunction may only lead to cognitive impairment in the context of underlying bipolar disorder-related neuropathology, such as neuroinflammation.^27^ In other words, the cognitive consequences of reduced glymphatic clearance might depend on the presence of bipolar disorder-specific neural vulnerabilities.^27^

Several study limitations would be addressed here. First, only the go/no-go task and DSST were used in the present study to examine the cognitive function in the participants. Additional neuropsychological assessments, such as the Wisconsin Card Sorting Test and the Stroop Color and Word Test, may be necessary to further clarify an association between glymphatic system function and cognitive function both in patients and healthy controls. Second, in order to prevent the clinical exacerbation, patients maintained their medications in the present study, which was more ethical and provided the natural data. Further studies with drug-free patients would be required to validate our findings. Third, the robustness of the ALPS index as a glymphatic system function marker has been reported in many previous studies.^4–7^ Alternative methods measuring the glymphatic system function, such as the dynamic contrast-enhanced MRI, would be applied to validate our and Ueda et al.’s findings.^6^ Fourth, the present study found no association between the ALPS index and insomnia symptoms, indicated by the HDRS insomnia subtotal scores, in patients with bipolar disorder, despite evidence suggesting the critical role of sleep in the glymphatic system function.^4^^,^^5^ The HDRS insomnia subtotal scores, as a clinical measurement, may not exactly reflect the sleep condition.^28^ Further studies using polysomnography to confirm the sleep condition would be necessary to clarify the association between glymphatic system function and sleep symptoms in patients with bipolar disorder. Fifth, the bipolar disorder subtypes, such as rapid cycling, were not assessed in the present study. Further studies would be required to elucidate the role of bipolar disorder subtypes in an association between glymphatic system function and cognitive function. Sixth, this study included only patients with bipolar disorder who scored ≤4 on the Clinical Global Impression rating scale, thereby limiting the generalizability of the findings to individuals with more severe symptomatology. Seventh, the participants enrolled in this study were relatively young (mean age ≈ 33 years), which may limit the generalizability of the findings to older populations. Furthermore, whether older individuals with bipolar disorder may exhibit stronger glymphatic–cognition associations due to aging effects remains to be elucidated. Finally, regarding the multiple comparison correction in associations between the glymphatic system function and cognitive function, the *P*-value was revised to 0.0125 (0.05/4). The associations between the ALPS index (*P*=.009), along with the diagnosis*ALPS index interaction (*P*=.002), and the DSST parameters in the entire sample remained significant, which still supported our findings that the association between the glymphatic system dysfunction and cognitive deficits was bipolar disorder-specific.

In conclusion, the detrimental impact of glymphatic system dysfunction on cognition—including processing speed, attention, working memory, and inhibitory control—appears to be context-dependent, specifically related to bipolar disorder. Notably, only patients with bipolar disorder who had a lower ALPS index (i.e., below the mean or within the lowest quartile) exhibited cognitive impairments, as assessed by the go/no-go task and the DSST, whereas healthy individuals with similarly low ALPS indices did not show such deficits. Conversely, patients with bipolar disorder who had a higher ALPS index (≥ mean or within the highest quartile) demonstrated cognitive performance comparable to that of healthy controls, suggesting a potentially protective role of preserved glymphatic system function in maintaining cognitive function. Further research would be warranted to elucidate the definite underlying pathomechanisms linking glymphatic system dysfunction to cognitive impairment in bipolar disorder and to investigate whether improving glymphatic function (eg, via sleep interventions) may mitigate cognitive deficits in bipolar disorder.

## Data Availability

The data that support the findings of this study are available on request from the corresponding author. The data are not publicly available due to the ethical regulation in Taiwan.
